# Down-regulation of *Risa* improves podocyte injury by enhancing autophagy in diabetic nephropathy

**DOI:** 10.1186/s40779-022-00385-0

**Published:** 2022-05-26

**Authors:** Pei-Pei Su, Dong-Wei Liu, Si-Jie Zhou, Hang Chen, Xian-Ming Wu, Zhang-Suo Liu

**Affiliations:** 1 Department of Nephrology, the First Affiliated Hospital of Zhengzhou University, Research Institutes of Nephropathy, Zhengzhou University, Zhengzhou, 450052 China; 2 Department of Nephrology and Rheumatology, the Third People’s Hospital of Zhengzhou, Zhengzhou, 450002 China; 3 Henan Province Research Center for Kidney Disease, Zhengzhou, 450052 China; 4 Key Laboratory of Precision Diagnosis and Treatment for Chronic Kidney Disease in Henan Province, Zhengzhou, 450002 China

**Keywords:** Diabetic nephropathy, LncRNA *AK044604*/Sirt1/GSK3β, Autophagy

## Abstract

**Background:**

LncRNA *AK044604* (regulator of insulin sensitivity and autophagy, *Risa*) and autophagy-related factors Sirt1 and GSK3β play important roles in diabetic nephropathy (DN). In this study, we sought to explore the effect of *Risa* on Sirt1/GSK3β-induced podocyte injury.

**Methods:**

Diabetic db/db mice received *Risa*-inhibition adeno-associated virus (AAV) via tail vein injection, and intraperitoneal injection of lithium chloride (LiCl). Blood, urine, and kidney tissue samples were collected and analyzed at different time points. Immortalized mouse podocyte cells (MPCs) were cultured and treated with *Risa*-inhibition lentivirus (LV), EX-527, and LiCl. MPCs were collected under different stimulations as noted. The effects of *Risa* on podocyte autophagy were examined by qRT-PCR, Western blotting analysis, transmission electron microscopy, Periodic Acid-Schiff staining, and immunofluorescence staining.

**Results:**

*Risa* and activated GSK3β were overexpressed, but Sirt1 was downregulated in DN mice and high glucose-treated MPCs (*P* < 0.001, db/m vs. db/db, NG or HM vs. HG), which was correlated with poor prognosis. *Risa* overexpression attenuated Sirt1-mediated downstream autophagy levels and aggravated podocyte injury by inhibiting the expression of Sirt1 (*P* < 0.001, db/m vs. db/db, NG or HM vs. HG). In contrast, *Risa* suppression enhanced Sirt1-induced autophagy and attenuated podocyte injury, which could be abrogated by EX-527 (*P* < 0.001, db/db + *Risa*-AAV vs. db/db, HG + *Risa*-LV vs. HG). Furthermore, LiCl treatment could restore GSK3β-mediated autophagy of podocytes (*P* < 0.001, db/db + LiCl vs. db/db, HG + LiCl vs. HG), suggesting that *Risa* overexpression aggravated podocyte injury by decreasing autophagy.

**Conclusion:**

*Risa* could inhibit autophagy by regulating the Sirt1/GSK3β axis, thereby aggravating podocyte injury in DN. *Risa* may serve as a therapeutic target for the treatment of DN.

**Supplementary Information:**

The online version contains supplementary material available at 10.1186/s40779-022-00385-0.

## Background

Diabetic nephropathy (DN) is a serious public health problem with an increasing incidence worldwide, but effective treatments are lacking. Approximately 40% of patients with diabetes develop the end-stage renal disease (ESRD) [1]. Therefore, it is urgent to find new therapeutic methods and targets.

Hyperglycemia-mediated alterations of extracellular and intracellular metabolism and nutrients, such as advanced glycation end products, increased protein kinase C activity, abnormal polyol metabolism [2], intracellular stress associated with renal hypoxia [3], mitochondrial reactive oxygen species [4], endoplasmic reticulum stress [5], and nutrient depletion are considered to be involved in the pathogenesis of DN. Autophagy is a highly conserved cellular process that degrades and recycles misfolded or dysfunctional proteins and damaged organelles to maintain cellular homeostasis via the lysosomal pathway [6]. Autophagy is regulated by nutritional status and intracellular stress, which are altered under diabetic conditions, and corresponding alterations in Sirtuin type-1 (Sirt1), Adenosine 5'-monophosphate-activated protein kinase (AMPK) and mammalian target of rapamycin (mTOR) autophagy pathways potentially exacerbate organelle dysfunction and lead to DN [7]. High glucose (HG) and nutrient abnormalities under diabetic conditions increase intracellular stress and inhibit autophagy by inhibiting Sirt1 and AMPK and activating mTOR, leading to the occurrence and progression of DN [2]. These findings indicate that hyperglycemia-induced alterations in autophagic activity are the key mechanism underlying diabetes-related podocyte injury.

Sirt1 belongs to a member of the silent information regulator 2 (Sir2)-like family of proteins [8, 9]. Sirt1 is involved in many physiological processes, such as metabolism, mitochondrial homeostasis, cell proliferation, autophagy, and apoptosis [8–11]. Sirt1 is a positive regulator of autophagy [2, 12]. Once activated, Sirt1 promotes autophagy by deacetylating autophagy-related proteins, such as autophagy-related factors (ATG)5, ATG7, Beclin-1, light chain 3 beta (LC3B), and p62 [13, 14]. In addition, Sirt1 can crosstalk with the AMPK and mTOR pathways and regulate energy metabolism and prosurvival pathways including autophagy [2]. Low expression of Sirt1 has been found in injured podocytes during DN [15, 16]. Moreover, specific knockdown of Sirt1 in podocytes induced severe podocyte damage and increased proteinuria, whereas kidney damage was significantly alleviated in diabetic mice with Sirt1 overexpression [9, 16]. Therefore, the expression of Sirt1 in podocytes influences the occurrence and development of DN.

Glycogen synthase kinase 3β (GSK3β) is a serine/threonine kinase and a key regulator of numerous cellular processes ranging from glycogen metabolism to cell cycle regulation and proliferation [17, 18]. GSK3β has complex and multitargeted biological effects, including regulation of multiple pathways involved in insulin resistance, oxidative stress, autophagy, and apoptosis, in DN-related podocyte injury [17–19]. GSK3β expression was increased in the podocyte HG environment. Lithium chloride (LiCl), as an inhibitor of GSK3β, alleviated HG-induced podocyte injury [20]. Increasing evidence has proven that nutritional energy imbalance caused by abnormal glucose metabolism leads to excessive autophagy inhibition and GSK3β activation, and GSK3β inhibition prevents ubiquitin proteome system-mediated degradation of mTOR mammalian targets, thereby enhancing autophagy [19, 21]. Therefore, GSK3β and autophagy play important roles in promoting DN podocyte injury and dysfunction.

Long non-coding RNAs (lncRNAs) have recently been confirmed to play key roles in the pathobiological process of diabetic kidney disease (DKD), including diabetic tubular disease and diabetic glomerular diseases such as podocytosis, endothelial dysfunction, mesangial lysis, and mesangial dilation. Many lncRNAs have been shown to be involved in DN podocyte damage. For example, lncRNA *TUG1* is related to the metabolic changes in podocytes in DN mice [22]. *MALAT1* is dysregulated during DN and participates in HG-induced podocyte injury through interactions with β-catenin [23]. Knockdown of *Gm5524* and overexpression of *Gm15645* triggered mouse podocyte apoptosis and decreased autophagy in HG culture conditions [24]. A transcript located very close to *Sirt1* gene, lncRNA *AK044604*, has been identified in the 10qb4 region in the mouse genome, and was named regulator of insulin sensitivity and autophagy (*Risa*) [25]. *Risa* can regulate insulin sensitivity and autophagy both in vivo and in vitro. Downregulation of *Risa* improves insulin sensitivity by enhancing autophagy in ob/ob mice [25].

In this study, we explored the expression of *Risa* in db/db mouse renal tissues and HG-induced mouse podocyte cells (MPCs), and predict the targets of *Risa* (miRNA or mRNA) by bioinformatics methods, which would be validated both in vitro and in vivo. We assumed that *Risa* could influence the downstream targets via the Sirt1-mediated autophagy pathway, thereby promoting DN progression. In short, our study sought to explore the mechanism underlying *Risa*/Sirt1/GSK3β-induced podocyte injury and reveal a potential target for DN screening and treatment.

## Methods

HG was used to mimic the diabetic environment and *Risa*-overexpression and *Risa*-inhibition MPC cell models were established. Normal glucose (NG) concentration group, high mannitol (HM) group, HG concentration group and HG + *Risa*-vehicle group were also included. Quantitative real-time polymerase chain reaction (qRT-PCR), Western blotting, immunofluorescence staining and transmission electron microscopy (TEM) were used to detect the mRNA and protein expressions of autophagy-associated factors (*Risa*, Sirt1, GSK3β, Beclin-1, LC3B and p62) and podocyte marker proteins (Nephrin, WT-1, NPHS2 and Desmin). The miRNAs that bound to *Risa* and GSK3β were validated by the double luciferase gene report assay. The levels of autophagy-related factors and podocyte proteins were examined in HG-induced MPCs cotransfected with *Risa* lentivirus (LV) and EX-527 (a Sirt1 inhibitor). LiCl was used as an autophagy activator to further observe the levels of autophagy-associated factors and podocyte proteins.

*Risa*-overexpression and *Risa*-inhibition diabetic db/db mouse models were established. Healthy control db/m mice and experimental control mice (db/db and db/db + *Risa*-vehicle) were also included. The mRNA and protein expression levels of autophagy-related proteins and podocyte proteins were detected by qRT-PCR, Western blotting, immunofluorescence staining, TEM and Periodic Acid-Schiff (PAS) staining. The levels of autophagy-associated proteins and podocyte proteins were detected in db/db mice intraperitoneally injected with EX-527 and LiCl. Blood and urine samples of mice in each group at different time points (6, 10, 14, 18 and 22 weeks) were collected to detect the changes in biochemical indexes [random blood glucose, urinary albumin-creatinine ratio (uACR), serum creatinine (Scr)]. Mice body weight changes were recorded.

### Animal studies

Four-week-old db/m (*n* = 20) and db/db experimental mice (*n* = 72) were purchased from the Institute of Model Animals of Nanjing University and maintained under standard conditions in the SPF animal room of the Institute of Medical Research of Zhengzhou University (Zhengzhou, Henan, China) for 2 weeks. The db/db mice and healthy control db/m mice were housed and maintained under a standard 12-h light/12-h dark cycle with ad libitum access to water and standard mouse chow. Animals were maintained under specific pathogen-free conditions and received humane care according to the criteria outlined in the National Institutes of Health (NIH) Guide [26]. This animal study was approved by the Institutional Animal Care and Use Committee of Zhengzhou University (2020-KY- 274).

The mice were randomly divided into five groups: (i) db/m group without any treatment (*n* = 20); (ii) db/db group without any treatment (*n* = 20); (iii) db/db + *Risa*-vehicle group (*n* = 20), mice received adeno-associated virus (AAV) empty vector treatment via tail-vein injection; (iv) db/db + *Risa*-AAV group (*n* = 20), mice received *Risa*-inhibition AAV via tail-vein injection; and (v) db/db + LiCl group (*n* = 12), db/db mice were intraperitoneally injected with LiCl [15 mg/(kg.qod)] for 4 weeks.

At 6, 10, 14, 18 and 22 weeks of age, mice’s body weights and random blood glucose level were measured, and urine samples were collected from db/m, db/db, db/db + *Risa*-vehicle and db/db + *Risa*-AAV groups at least three times on the same day. Then, 4 mice were killed randomly in each group, and renal tissues and serum samples were taken. At the age of 10 weeks, 4 mice in the db/m, db/db, db/db + *Risa-*vehicle and db/db + *Risa-*AAV groups without any treatment were killed randomly and used as the treatment baseline controls. The remaining mice in the db/db + *Risa*-vehicle group and db/db + *Risa*-AAV group were injected with empty vector or *Risa*-inhibition AAV through the visual tail vein method. The total titre of AAV after packaging was 3.8 × 10^12^ (v.g./ml), and the dose for each mouse was 2 × 10^10^ (v.g./ml). At the age of 14 weeks, empty vector and *Risa*-AAV were injected again at the same dose into the mice in the db/db + *Risa-*vehicle and db/db + *Risa*-inhibition AAV groups. At the ages of 10, 14, 18, 22 weeks, db/db mice were intraperitoneally injected with LiCl. The mice’s blood, urine and kidney samples were collected and detected by qRT-PCR, Western blotting analysis, TEM, PAS staining and confocal immunofluorescence staining and the relevant data (blood glucose, body weight, Scr, and uACR) were also analyzed. The detailed experimental processes are in the Additional file 1.

According to the manufacturer's instructions (GeneChem Co., Ltd, Shanghai, China), the AAV vector that interfered with *Risa* was constructed. The target sequence was GGAAAGATCTGGCGATTGA, and the vector was U6-MCS-CAG-mCherry, serotype 9. *Risa* was amplified by PCR using specific primers (forward: 5′-TCTGGA GAGCCCAACCT-3′, reverse: 5′-TCCTTCAAACGCGAGAGAG-3′). *Risa*-inhibition AAV helper-free system consisted of three plasmids: virus vector, pAAV-RC vector and pHelper vector. The packaging process was as follows: (1) the target fragment was cloned into the vector, (2) HEK293T cells were transfected with the packaging plasmid, (3) the viruses were harvested and purified by centrifugation and ultrafiltration. Finally, the virus titre was determined by qRT-PCR.

### Cell culture and transfection

Immortalized MPCs were donated by academician Zhi-Hong Liu of Nanjing University School of Medicine (Nanjing, China). MPCs were immediately placed into a water bath at 37 °C with gentle shaking after the liquid nitrogen evaporated. When completely melted, the cryopreservation-containing cells were transferred to a sterilized 15 ml centrifuge tube and centrifuged for 10 min at 1000 rpm after the supernatant was removed, the cells were resuspended in the complete culture medium and inoculated in a culture dish with 33 ℃ culture medium (RPMI 1640 medium containing 10% FBS, 1% penicillin/streptomycin solution, 4 ng/ml γ-interferon, and 0.4‰ Mycoplasma scavenger). The cells were evenly distributed through gentle shaking and then placed in an incubator. The culture conditions were set at 33 ℃ with 5% CO_2_ under saturated humidity. The medium was changed the next day, and cell growth was observed. The morphology of normal podocytes observed under a microscope was round or quasi round, the nucleus was located in the center, and the foot process was short and thick or missing. When cell confluence reached 70–80%, the cells were subcultured at a ratio of 1:3. The medium was changed, the configured 37 ℃ medium (RPMI 1640 medium containing 10% FBS, 1% penicillin/streptomycin mixed solution, and 0.4‰ Mycoplasma scavenger) was added, and the MPCs were transferred to a 37 ℃ incubator. MPCs continued to grow, differentiate and mature in the same RPMI medium without γ-interferon at 37 ℃. Microscopically, the mature podocytes became irregular, and stretched out a long bifurcation. After 10–14 d of differentiation and maturation, HM, HG, LiCl, EX-527 and other stimuli were added to the 37 °C culture medium and the cells were divided into the following groups: (i) NG, 5.6 mmol/L glucose; (ii) HM, 5.6 mmol/L glucose + 44.4 mmol/L mannitol; (iii) HG, 30 mmol/L glucose; (iv) HG + LiCl (30 mmol/L); (v) HG + *Risa*-LV; (vi) HG + *Risa*-vehicle; and (vii) HG + *Risa*-LV + EX-527 (10 μmol/L). The cells were collected 48 h after stimulation for subsequent tests. To explore the time gradient of autophagy stimulated by HG, the cells were collected at 0, 12, 24, 48, 72 and 96 h after HG stimulation for Western blotting and immunofluorescence tests. LiCl was purchased from Beijing DingGuo Changsheng Biotechnology (Beijing, China) and EX-527 was purchased from Beijing Bioss Biotechnology (Beijing, China).

Lentivirus vectors that interfered with *Risa* were constructed according to the manufacturer's instructions (GeneChem Co., Ltd., Shanghai, China). The target sequence was GGAAAGATCTGGCGATTGA, and the vector was U6-MCS-CAG-mCherry, serotype 9. The *Risa*-inhibition lentivirus vector system consisted of the following three plasmids: GV lentivirus vector series, pHelper 1.0 vector and pHelper 2.0 vector. The packaging process was as follows: (1) the three plasmids were cotransfected into HEK293T cells, (2) the virus was harvested 48–72 h after transfection, (3) the samples were centrifuged to remove impurities, and (4) the samples were concentrated. Finally, the virus titre was examined by qRT-PCR. Preliminary experiments were carried out to obtain the multiplicity of infection values (MOI = 50), and then the virus was transfected into MPCs. Finally, the virus was killed by puromycin to obtain the stable transformation strain.

### Luciferase activity assay

Plasmids of m-*GSK3β*-3′UTR or m-*AK044604* containing the wild-type binding sites and mmu-miR-6380/mmu-miR-706/mmu-miR-1195/mmu-miR-3089-5p were cotransfected into HEK293T cells with Luciferase Assay Reagent (Hanbio Co., Ltd., Shanghai, China). Luciferase activity was analyzed using the Promega Dual-Luciferase system (Promega Biotech Co., Ltd., Beijing, China). Double luciferase reporter genes were constructed by Hanbio Biotechnology Co., Ltd.

### RNA extraction and qRT-PCR

Tissue and cell total RNA were extracted using TRIzol reagent (Thermo Fisher Scientific, Shanghai, China). Reverse transcription was performed using a First Strand cDNA Synthesis Kit. The polymerase chain reaction was performed using a synergy brands (SYBR) Green qPCR kit (Thermo Fisher Scientific, Shanghai, China). Relative gene expression data were calculated using the comparative threshold cycle method with glyceraldehyde-3-phosphate dehydrogenase (GAPDH, Servicebio Co., Ltd., Wuhan, China) as the housekeeping gene. All assays were run in triplicate. The primers for each gene are shown in Additional file 2: Table S1.

### Western blotting assay

Total protein lysates were extracted from mouse renal cortex tissue or cells using radio immunoprecipitation assay (RIPA) lysis buffer (Solarbio Co., Ltd., Beijing, China). Protein samples were separated by 10–12% sodium dodecyl sulphate–polyacrylamide gel electrophoresis (SDS-PAGE) and transferred to a polyvinylidene fluoride membrane. After blocking with 5% skim milk in phosphate-buffered saline with 0.1% Tween 20 for 1 h, the membranes were incubated with the primary antibody at 4 °C overnight and then with the secondary antibody at room temperature for 1 h. A High Sig ECL Western blotting substrate development kit was used to develop the blot. The relative grey intensity of each target protein expression band was quantitatively analyzed by ImageJ, and GAPDH was used as an internal reference. The ratio of each target protein to GAPDH was taken as the relative expression level of the target protein. The primary antibodies are shown in Additional file 2: Table S2.

### TEM

The kidney specimens or MPCs were fixed in glutaraldehyde and then sent for electron microscopy. Ultrathin sections were cut with an ultramicrotome, stained with 2% uranyl acetate and lead citrate, and examined with a JEOL JEM-1400 Plus transmission electron microscope. Autophagosomes in the podocytes were identified under 5000 × and 25,000 × high-power fields. Each sample was examined and ten podocytes were randomly selected to count the number of autophagosomes and autophagolysosomes in podocytes. After counting, statistical analysis was performed by an experienced pathologist who also served as a nephrologist.

### Renal histology

Formalin-fixed and paraffin-embedded kidney tissues were prepared as 4 μm sections and deparaffinized and hydrated. Sections were processed for periodic acid-Schiff staining. Slides were rinsed in distilled water after oxidizing in 0.5% periodic acid for 5 min, incubated in Schiff reagent for 15 min, washed in lukewarm tap water for 5 min, and counterstained in Mayer’s haematoxylin.

### Confocal immunofluorescence staining and analysis

Kidney tissue sections or cells were fixed using cold methanol for 40 min and incubated overnight at 4 ℃ with primary antibodies. Secondary antibodies conjugated with Alexa Fluor® 488 or 594 were incubated at 25 ℃ for 1 h. Finally, sections were counterstained with propidium iodide (PI) or 4′,6-diamidino-2-phenylindole (DAPI) and visualized under a Zeiss LSM 880 confocal microscope. The primary antibodies are shown in Additional file 2: Table S3.

### Statistical analyses

Statistical analyses were performed using SPSS (version 20, IBM Corp, Armonk, NY, US) or GraphPad Prism (version 9.0, GraphPad Software Inc., San Diego, CA, US) software. Data are expressed as means ± standard deviation (SD, unless otherwise stated). Comparison between two or more groups were performed by one-way analysis of variance (ANOVA). *P-*value < 0.05 in two-tailed tests were considered statistically significant in all analyses. All results were repeated at least three times.

## Results

### Effect of *Risa* overexpression and inhibition on HG-induced podocyte injury

**Fig. 1:**
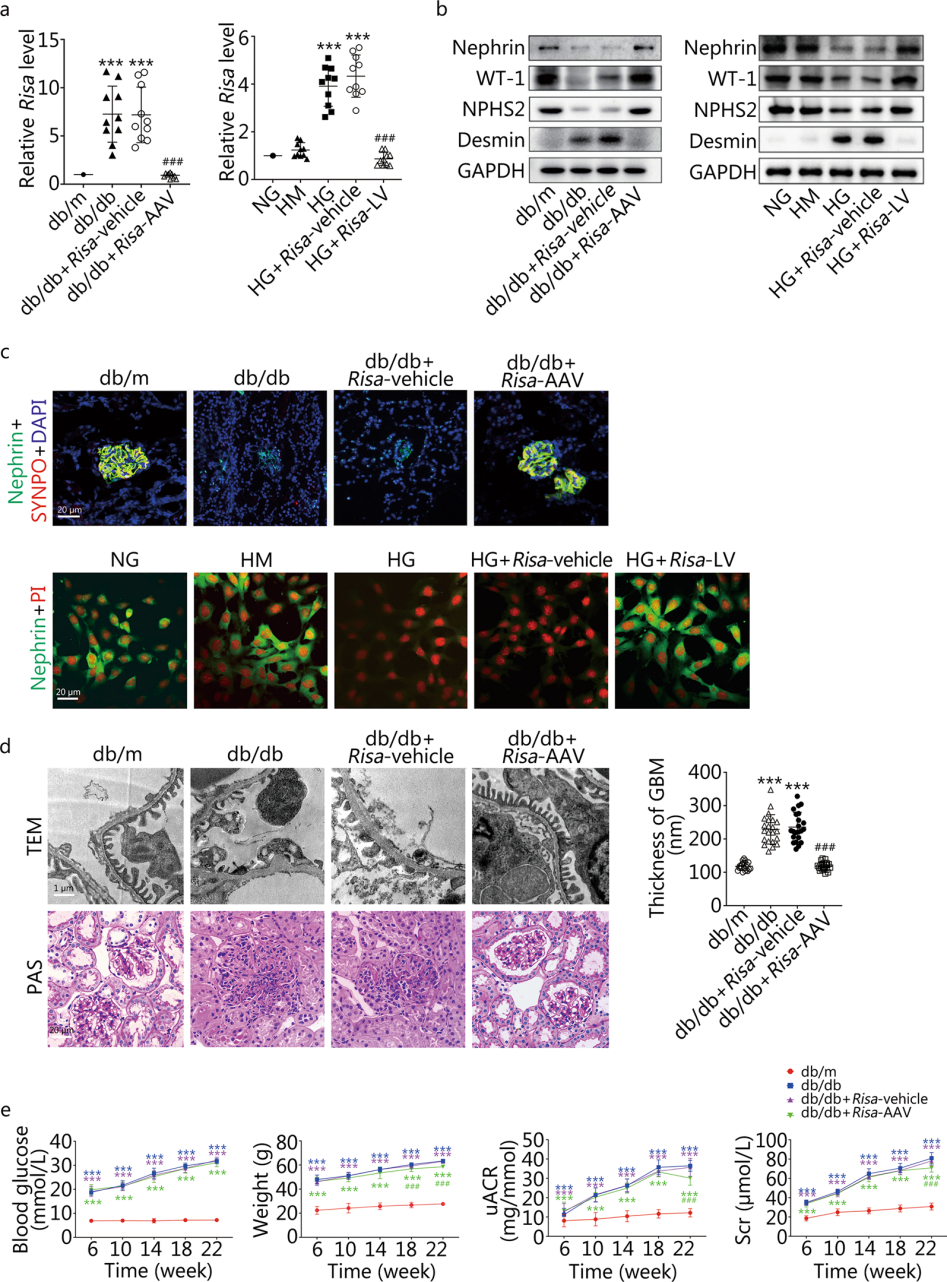
Effect of *Risa* overexpression and *Risa* inhibition on HG-induced podocyte injury. **a** Expression of *Risa* in DN mice and MPCs was examined by qRT-PCR (*n* = 10). **b** Expression of Nephrin, WT-1, NPHS2, and Desmin was measured by Western blotting in the mouse groups at 22 weeks and the MPC groups under different stimulations. **c** Immunofluorescence staining analysis of Nephrin (green) and/or Synaptopodin (red) expresssion, the nuclei were stained with DAPI (blue) or PI (red). **d** Thickness of the GBM was calculated under TEM (*n* = 22), and the morphology of kidney was observed by PAS staining in the db/m, db/db, db/db + *Risa*-vehicle and db/db + *Risa*-AAV groups at 22 weeks. **e** Changes in random blood glucose, weight, uACR, and Scr in the mouse groups at different time points. Values are expressed as mean ± SD of three independent experiments. ^***^P < 0.001 vs. db/m group or NG and HM groups, ^###^*P* < 0.001 vs. db/db and db/db + *Risa*-vehicle groups or HG and HG + *Risa*-vehicle groups. SYNPO synaptopodin, PAS periodic acid-schiff stain, MPCs mouse podocyte cells, *Risa* lncRNA *AK044604*, AAV adeno-associated virus, GAPDH glyceraldehyde-3-phosphate dehydrogenase, GBM glomerular basement membrane, HG high glucose, HM high mannitol, LV lentivirus, NG normal glucose, TEM transmission electron microscopy

We initially determined *Risa* expression in DN mice or MPCs following different interventions. The qRT-PCR results showed that *Risa* expression in diabetic db/db mice was significantly higher than that in healthy controls (*P* < 0.001, db/m vs. db/db or db/db + *Risa*-vehicle), but it decreased after *Risa*-AAV treatment (*P* < 0.001, db/db + *Risa*-AAV vs. db/db or db/db + *Risa*-vehicle) (Fig. 1a). Similarly, the expression of *Risa* in the HG group was increased significantly compared with that in the NG and HM groups (*P* < 0.001, NG, HM vs. HG or HG + *Risa*-vehicle), but it was decreased after *Risa*-LV treatment (*P* < 0.001, HG + *Risa*-LV vs. HG or HG + *Risa*-vehicle) (Fig. 1a). The qRT-PCR results confirmed the successful overexpression and inhibition of *Risa* in diabetic db/db mice and HG-induced MPCs. We then examined the effect of *Risa* overexpression and inhibition on podocyte injury. Western blotting and immunofluorescence staining results supported that *Risa* overexpression in the diabetic db/db mice or HG-treated MPCs resulted in poor expression levels of podocyte proteins (Fig. 1b, c). The levels of podocyte protective markers (Nephrin, Synaptopodin, WT-1, and NPHS2) in db/db mice or HG-induced MPCs were significantly decreased compared with the db/m group or NG and HM groups (Fig. 1b, c), but the opposite trend was observed for Desmin (Fig. 1b). However, the levels of podocyte proteins were increased markedly after *Risa*-AAV or *Risa*-LV treatment (Fig. 1b, c). Moreover, TEM results showed that the thickness of the glomerular basement membrane (GBM) in db/db mice was significantly increased (*P* < 0.001, db/m vs. db/db or db/db + *Risa*-vehicle), and the number of foot processes was reduced in db/db mice, but GBM thicknesses was decreased after *Risa*-AAV treatment (*P* < 0.001, db/db + *Risa*-AAV vs. db/db or db/db + *Risa*-vehicle), and the foot processes were repaired (Fig. 1d). PAS staining results revealed that abnormal kidney morphology was obviously aggravated in db/db mice, but matrix hyperplasia and cyst cavities were markedly improved after *Risa*-AAV treatment (Fig. 1d). Moreover, we observed that the levels of blood glucose, body weight, uACR, and Scr in db/db mice were significantly higher than those in db/m mice (*P* < 0.001, Fig. 1e). The body weight, uACR and Scr levels (except blood glucose) were significantly decreased after *Risa*-AAV treatment of db/db mice compared with the db/db control mice at 22 weeks (*P* < 0.001, db/db + *Risa*-AAV vs. db/db or db/db + *Risa*-vehicle) (Fig. 1e). Notably, HG-induced podocyte injury and renal function damage were counteracted by *Risa*-inhibition but aggravated by *Risa*-overexpression. Taken together, *Risa* blockade abrogated the synergistic effects of *Risa*-overexpression on podocyte injury and renal function damage.

### Effect of *Risa* overexpression and inhibition on HG-induced autophagy damage

**Fig. 2:**
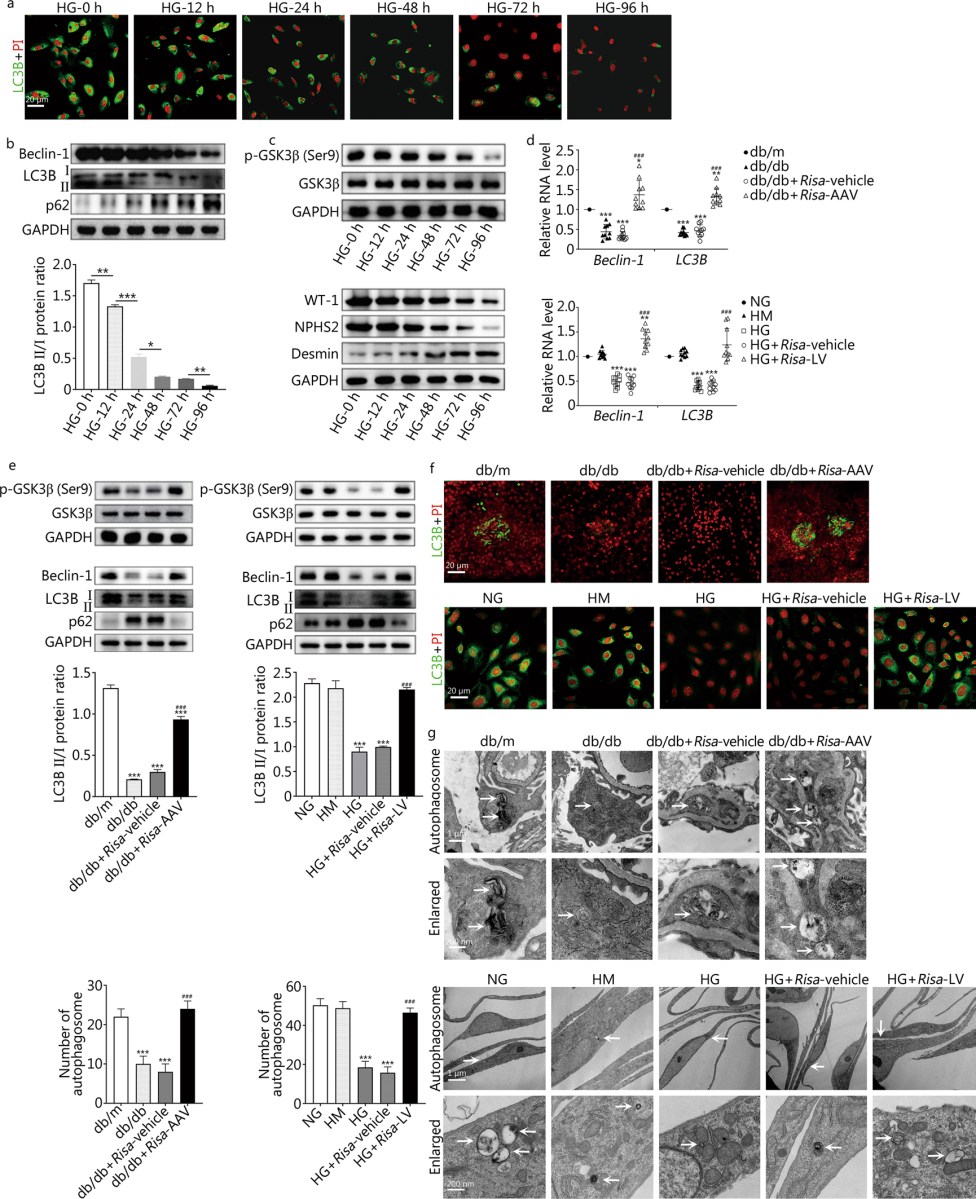
Effect of *Risa* overexpression and *Risa* inhibition on HG-induced podocyte autophagy damage. **a** Expression of LC3B was detected by immunofluorescence at different observation times in HG-induced MPCs. **b** Expression of LC3B, Beclin-1 and p62 was detected by Western blotting. The bar graph shows the quantification of LC3B II/I. **c** Expression of GSK3β, p-GSK3β, WT-1, NPHS2 and Desmin was detected by Western blotting. **d** Expression of *Beclin-1* and *LC3B* was examined by qRT-PCR in the mouse groups and MPC groups (*n* = 10). **e** Expression of GSK3β, p-GSK3β, Beclin-1, LC3B and p62 was examined by Western blotting. The bar graph shows the quantification of LC3B II/I. **f** Immunofluorescence staining analysis of LC3B (green) expression, the nuclei were stained with PI (red). **g** Autophagosomes (white arrows) were observed by TEM. The mean numbers of autophagosomes were counted. Values are expressed as mean ± SD of three independent experiments. ^*^P < 0.05, ^**^P < 0.01, ^***^P < 0.001 vs. the previous time group or db/m group or NG and HM groups, ^###^*P* < 0.001 vs. db/db and db/db + *Risa*-vehicle groups or HG and HG + *Risa*-vehicle groups. MPCs mouse podocyte cells, *Risa* lncRNA *AK044604*, AAV adeno-associated virus, GAPDH glyceraldehyde-3-phosphate dehydrogenase, GSK3β glycogen synthase kinase 3β, HG high glucose, HM high mannitol, LV lentivirus, NG normal glucose

To determine whether autophagy attenuation is involved in the destructive effect of *Risa* on podocytes, MPC autophagy was assessed by immunofluorescence staining of LC3B (Fig. 2a) and by Western blotting analysis of LC3B, Beclin-1, p62 (Fig. 2b) and GSK3β (Fig. 2c) and WT-1, NPHS2 and Desmin (Fig. 2c) at different observation times and under different stimulations. The levels of podocyte autophagy-associated factors (Beclin-1, LC3B, p-GSK3β, WT-1 and NPHS2), as well as the LC3B II/I ratio (*P* = 0.007, HG-12 h vs. HG-0 h; *P* < 0.001, HG-24 h vs. HG-12 h; *P* = 0.020, HG-48 h vs. HG-24 h; *P* = 0.082, HG-72 h vs. HG-48 h; *P* = 0.004, HG-96 h vs. HG-72 h) (Fig. 2b) decreased with time after HG stimulation and decreased significantly after 24 h (Fig. 2a–c). The opposite trend was observed for p62 and Desmin (Fig. 2b, c). Therefore, podocyte autophagy decreased gradually with the prolongation of HG stimulation. We then examined the effect of *Risa*-overexpression and *Risa*-inhibition on podocyte autophagy. The qRT-PCR results in vivo and in vitro showed that the expressions of *Beclin-1* and *LC3B* in db/db mice and HG-mediated MPCs were significantly lower than those in db/m group or NG and HM groups (*P* < 0.001, db/m vs. db/db or db/db + *Risa*-vehicle; NG, HM vs. HG or HG + *Risa*-vehicle) but increased after *Risa*-AAV or *Risa*-LV treatment (*P* < 0.001, db/db + *Risa*-AAV vs. db/db or db/db + *Risa*-vehicle; HG + *Risa*-LV vs. HG or HG + *Risa*-vehicle) (Fig. 2d). Western blotting results in vivo and in vitro also showed that the expressions of p-GSK3β, Beclin-1, LC3B, as well as the LC3B II/I ratio in db/db mice and HG-mediated MPCs were significantly lower than those in db/m group or NG and HM groups (*P* < 0.001, db/m vs. db/db or db/db + *Risa*-vehicle; NG, HM vs. HG or HG + *Risa*-vehicle) but increased after *Risa*-AAV or *Risa*-LV treatment (*P* < 0.001, db/db + *Risa*-AAV vs. db/db or db/db + *Risa*-vehicle; HG + *Risa*-LV vs. HG or HG + *Risa*-vehicle) (Fig. 2e). The trend of LC3B expression based on immunofluorescence staining was the same as the qRT-PCR and Western blotting results in both db/db mice and HG-induced MPCs (Fig. 2f). In addition, TEM results revealed that the number of autophagosomes in db/db mice and HG-induced MPCs were lower than that in db/m group or NG and HM groups (*P* < 0.001, db/m vs. db/db or db/db + *Risa*-vehicle; NG, HM vs. HG or HG + *Risa*-vehicle) but increased after *Risa*-AAV or *Risa*-LV therapy (*P* < 0.001, db/db + *Risa*-AAV vs. db/db or db/db + *Risa*-vehicle; HG + *Risa*-LV vs. HG or HG + *Risa*-vehicle) (Fig. 2g). A diabetic environment or HG enhanced the inhibition of the protein levels of LC3B, Beclin-1, p-GSK3β; LC3B II/I ratio; and the number of autophagosomes, while the opposite trend was observed for p62. Thus, the damage to podocyte autophagy induced by HG was counteracted by *Risa*-inhibition but aggravated by *Risa*-overexpression. We also found that *Risa*-inhibition abrogated the synergistic effect of *Risa*-overexpression on autophagy damage.

### *Risa* aggravated HG-induced podocyte injury by reducing autophagy

**Fig. 3:**
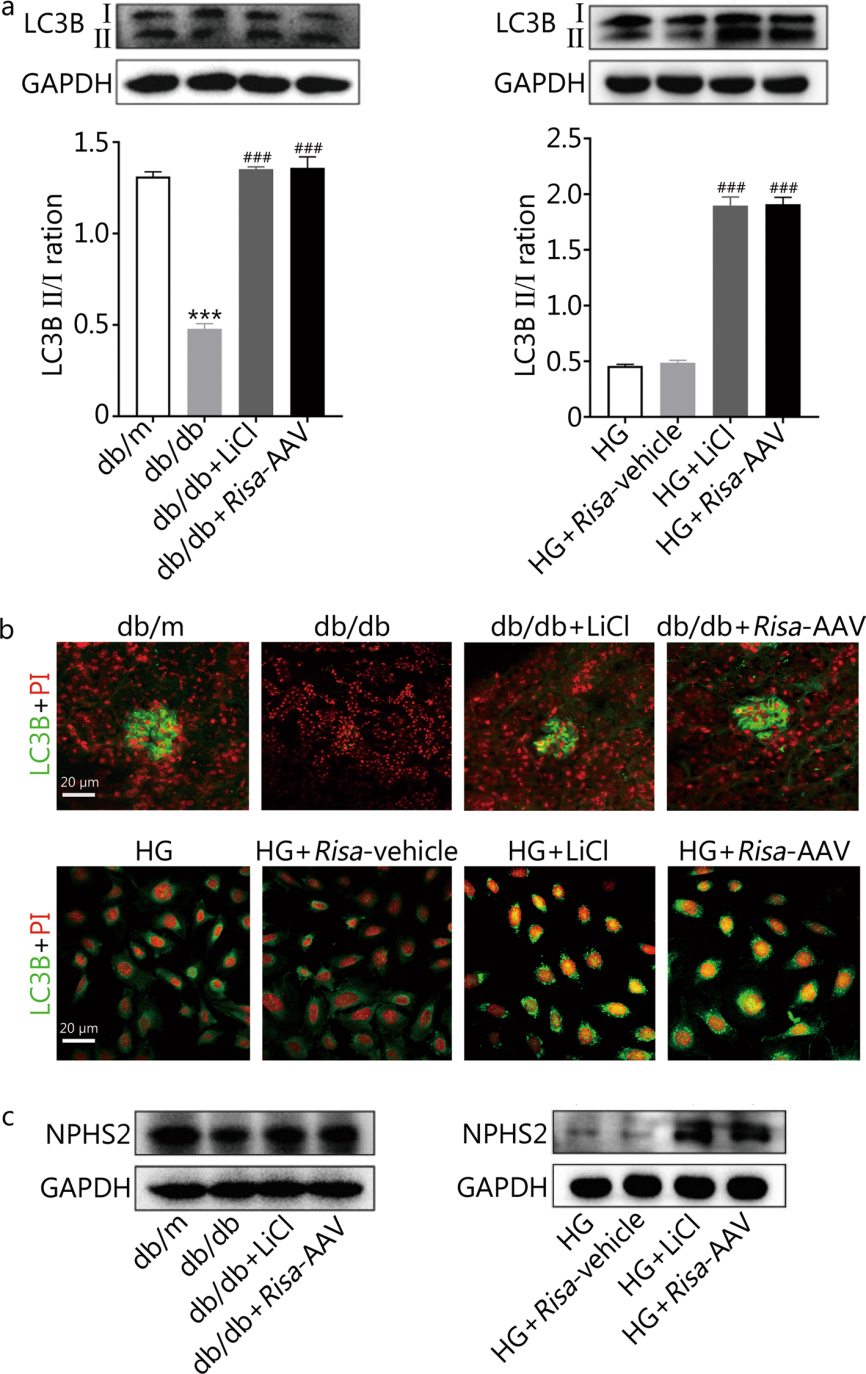
Effect of *Risa* overexpression and *Risa* inhibition on HG-induced podocyte autophagy damage. The level of LC3B was detected by Western blotting (**a**) and immunofluorescence (**b**) in the mouse groups and MPC groups. The bar graph shows the quantification of LC3B II/I. **c** Level of NPHS2 was detected by Western blotting. Values are expressed as mean ± SD of three independent experiments. ^***^P < 0.001 vs. db/m group, ^###^*P* < 0.001 vs. db/db group or HG and HG + *Risa*-vehicle groups. MPCs mouse podocyte cells, *Risa* lncRNA *AK044604*, AAV adeno-associated virus, GAPDH glyceraldehyde-3-phosphate dehydrogenase, HG high glucose

The above research conclusions indicated that *Risa* expression was increased and podocyte autophagy damage was more severe under HG conditions, and *Risa* inhibition could improve podocyte and autophagy damage. To verify the involvement of autophagy pathway, podocyte autophagy was observed after treatment with LiCl (an activator of autophagy, also an inhibitor of GSK3β) in db/db mice or HG-induced MPCs. Western blotting and immunofluorescence staining results showed that LiCl treatment increased the levels of LC3B and NPHS2 in db/db mice and HG-induced MPCs (Fig. 3a–c), as well as the LC3B II/I ratio (*P* < 0.001, db/db + LiCl vs. db/db; HG + LiCl vs. HG or HG + *Risa*-vehicle) (Fig. 3a). Of note, LiCl treatment abrogated the effect of *Risa* on autophagy in DN mice and HG-induced MPCs to a certain extent, reversing *Risa*-overexpression-mediated podocyte injury. These results implied that *Risa* aggravated HG-induced podocyte injury by reducing autophagy.

### *Risa* suppressed autophagy and aggravated podocyte injury via Sirt1-mediated autophagy axis

To explore the relationship between *Risa* and HG-induced podocyte autophagy pathway, we conducted bioinformatics analysis and the results revealed putative binding sites between *Risa*, *GSK3β* and miRNAs (miR-6380, miR-706, miR-1195 and miR-3098-5p) (Additional file 2: Fig. S1a, b). We transfected miRNA mimics into HEK293T cells. Double luciferase reporting results showed that mmu-miR-6380/mmu-miR-706/mmu-miR-1195/mmu-miR-3098-5p significantly downregulated the luciferase activity of m-*AK044604*-wt (*P* < 0.001, Additional file 2: Fig. S1c). In contrast, there were no significant differences in luciferase activity after cotransfection with mmu-miRNAs and m-*GSK3β*-3′UTR-wt (*P* > 0.05, Additional file 2: Fig. S1d).

**Fig. 4:**
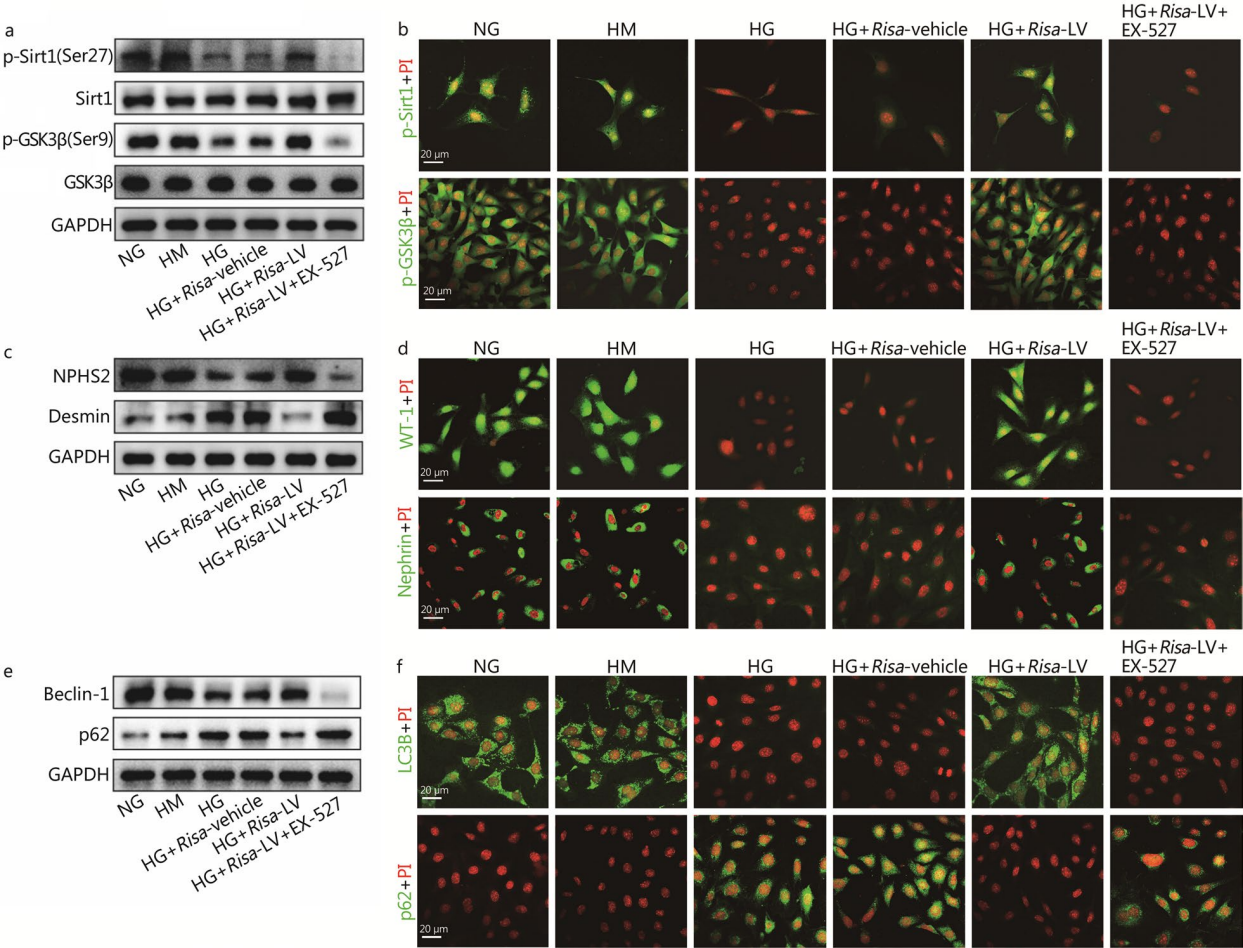
*Risa* suppresses autophagy and aggravats podocyte injury via Sirt1-autophagy axis. The HG-induced MPCs were cotransfected with *Risa*-LV and EX-527. The levels of phosphorylated Sirt1 and GSK3β were examined by Western blotting (**a**) and immunofluorescence staining (**b**). **c** Expression of NPHS2 and Desmin was assessed by Western blotting. **d** Expression of WT-1 and Nephrin was observed by immunofluorescence staining. **e** Levels of Beclin-1 and p62 were examined by Western blotting. **f** Levels of LC3B and p62 were assessed by immunofluorescence staining. MPCs mouse podocyte cells, *Risa* lncRNA *AK044604*, GAPDH glyceraldehyde-3-phosphate dehydrogenase, GSK3β glycogen synthase kinase 3β, HG high glucose, HM high mannitol, NG normal glucose, LV lentivirus, Sirt1 sirtuin 1

We supposed that there were interactions between *Risa* and Sirt1 in the process of DN, and the crosstalk between *Risa* and autophagy might be a therapeutic target for preventing and treating DN. However, the precise underlying molecular mechanisms were not fully understood. We speculated that *Risa* could bind to RNA or protein in a HG environment and decrease the levels of downstream autophagy-related proteins (GSK3β, Beclin-1, and LC3B) through Sirt1, leading to podocyte damage. Thus, we used EX-527 (a Sirt1 inhibitor) to observe the changes of podocyte autophagy in the *Risa*-inhibition db/db mice model and HG-induced MPCs. Western blotting and immunofluorescence staining results showed that EX-527 greatly decreased the levels of phosphorylated Sirt1 and GSK3β in MPCs with HG-induced *Risa*-inhibition (Fig. 4a, b). Furthermore, the levels of podocyte markers (NPHS2, WT-1, and Nephrin) were significantly decreased after transfecting EX-527 into MPCs with HG-induced *Risa*-inhibition, but the opposite result was found for Desmin expression (Fig. 4c, d). In addition, the levels of autophagy markers (Beclin-1 and LC3B) were notably downregulated, whereas the p62 level was significantly upregulated in HG-induced *Risa*-inhibition MPCs transfected with EX-527 (Fig. 4e, f). More importantly, *Risa* inhibition effectively abolished *Risa*-mediated autophagy inhibition and podocyte injury induced by HG, while Sirt1 inhibition abrogated the autophagy-promoting and anti-injury effects of *Risa*-inhibition in HG-treated MPCs. Therefore, *Risa* attenuated Sirt1-mediated downstream autophagy-related protein levels and aggravated podocyte injury by inhibiting the expression of Sirt1 in HG-induced MPCs. Taken together, *Risa* suppressed autophagy and aggravated podocyte injury via the Sirt1-autophagy axis under HG conditions.

## Discussion

DN is generally considered a podocyte disease characterized by podocyte injury after glomerular filtration barrier rupture, which results in proteinuria and renal failure [27]. Abnormal autophagy is an important cause of DN. In the context of DKD, whole gene transcriptome analysis has confirmed the existence of a variety of pathways involved in autophagy abnormalities. In addition, abnormal autophagy has been suggested to be an important catalyst involved in the progression of DN to ESRD [28, 29]. Our results revealed that the *Risa* transcript, which regulates insulin sensitivity and autophagy function, was overexpressed in DN models. Moreover, we found that the transcriptional level of GSK3β was down-regulated by Sirt1, and the autophagic flux of podocytes was reduced, resulting in proteinuria and glomerular damage. However, we also discovered that *Risa* inhibition could reverse the adverse effects of *Risa* on podocyte autophagy injury and glomerular damage. To the best of our knowledge, this is the first study reporting on the mechanism of *Risa*/Sirt1/GSK3β-mediated autophagy abnormalities in podocyte injury in DN, including the beneficial effect of *Risa* inhibition on the DN background.

*Risa* is located close to the Sirt1 locus and is initiated in a divergent fashion from the Sirt1 promoter. Both Sirt1 and *Risa* are transcribed by RNA polymerase II, suggesting that *Risa* is closely related to Sirt1 [25]. However, the specific mechanism of regulation remains unclear. To date, studies have attempted to investigate the regulatory mechanism of *Risa* on insulin resistance and autophagy pathway. The results have shown that the overexpression of *Risa* in mouse primary hepatocytes or C2C12 myotubes can reduce insulin-stimulated phosphorylation of insulin receptor, protein kinase B (Akt), and GSK3β, as well as the downstream autophagic flux marker of LC3B [25]. In contrast, the knockout of *Risa* can reverse the above adverse effects, suggesting that *Risa* regulates insulin sensitivity by affecting autophagy [25]. In addition, a study has confirmed that diabetes-induced downregulation of Sirt1 leads to reduced autophagy and accelerated risk of DN [30]. Thus, to explore the mechanism of *Risa* regulation in podocyte autophagy in DN, we searched for miRNAs or mRNAs associated with *Risa* and evaluated the regulatory pathway of *Risa* in vivo and in vitro. We observed impaired autophagy activity, podocyte injury, proteinuria, and glomerular damage in the DN model. These findings might be related to *Risa*/Sirt1/GSK3β-mediated autophagy pathway. In db/db mice or HG-induced podocytes, *Risa* expression was significantly increased; *Risa* overexpression inhibited the phosphorylation of Sirt1 at Ser27, reduced the activity and expression of Sirt1, decreased the phosphorylation of GSK3β at Ser9 (the total GSK3β remained unchanged; thus, the activation of GSK3β increased), impaired autophagic flux such as LC3B and led to renal podocyte injury. In contrast, *Risa* inhibition increased the expression of Sirt1, reduced the activation of GSK3β, restored the podocyte autophagy level, and improved renal injury and proteinuria. Thus, we concluded that *Risa* inhibition had a protective role in DN by indirectly restoring podocyte autophagy function, repairing podocyte injury, reducing proteinuria, and improving glomerular damage; however, this protective effect could be reversed by Sirt1 inhibitor (EX-527). Furthermore, LiCl treatment restored the autophagy function of podocytes. Therefore, *Risa* could cause podocyte injury in DN by inhibiting Sirt1/GSK3β-mediated autophagy. This observation may provide valuable insight into the molecular mechanisms of *Risa* action.

**Fig. 5:**
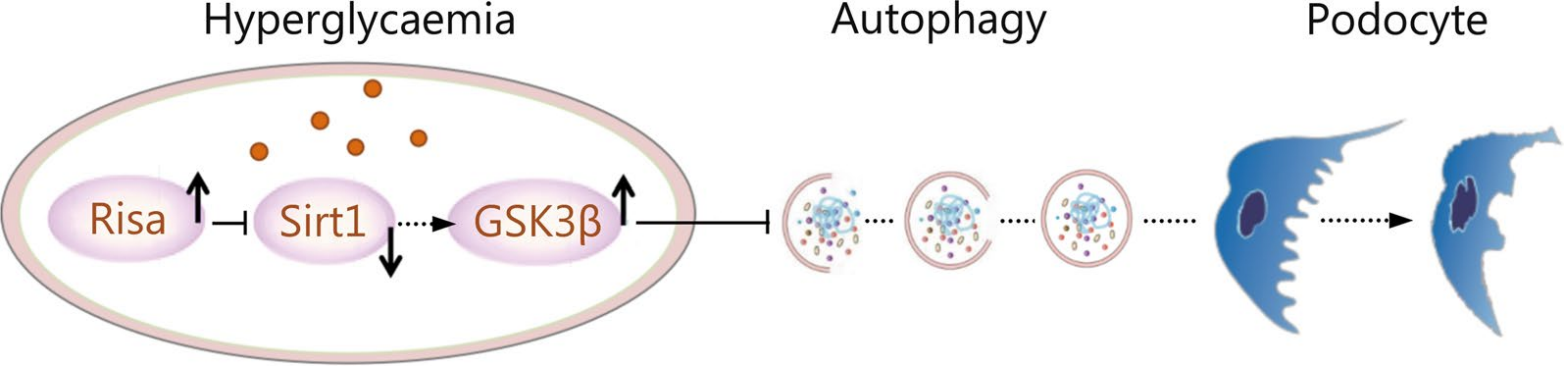
A working model for *Risa* regulation on podocyte injury and autophagy in DN Under hyperglycemia, the activity and expression of *Risa* increased. *Risa* overexpression inhibited the level of Sirt1, increased the activation of GSK3β, and then changed the expression and function of autophagy-related proteins and podocyte proteins through interaction and phosphorylation, which eventually led to podocyte damage. DN diabetic nephropathy, *Risa* lncRNA *AK044604*, Sirt1 sirtuin 1, GSK3β glycogen synthase kinase 3β

Autophagy plays a crucial role in the pathogenesis of DN [2]. During autophagy, a double-membrane-bound vesicle, termed an autophagosome, forms in the cytosol, sequesters cytoplasm or targeted cargoes, and fuses with the lysosome, releasing the inner vesicle and the autophagic body into the lumen [31, 32]. The cargoes are broken down by resident hydrolases, and the resulting macromolecules are recycled [31, 32]. It is well recognized that lncRNA regulation of cellular processes partially depends on lncRNA cellular localization [33]. Nuclear lncRNAs are enriched for functionality involving chromatin interactions, transcriptional regulation, and RNA processing, while cytoplasmic lncRNAs can modulate mRNA stability or translation and influence cellular signaling cascades [33, 34]. LncRNA modulation of RNA metabolism is an emerging theme for lncRNAs that are enriched in the cytoplasm, where the lncRNAs participate in cellular biological processes by functioning as competing endogenous RNAs (ceRNAs) or “RNA sponges” to regulate mRNA stability, mRNA alternative splicing, and protein localization [33]. In this study, we reported that *Risa* could antagonize the Sirt1-mediated GSK3β pathway in DN, thereby inhibiting podocyte autophagy. The regulatory mechanism of *Risa* action is summarized in Fig. 5. *Risa* is localized preferentially in the cytoplasm [25]. Sirt1 and GSK3β are located in the nucleus and/or cytoplasm, mainly in the nucleus, and nuclear Sirt1 may undergo cytoplasmic autophagy-mediated lysosomal degradation during the cellular process [35]. In accordance with the above statements, these results suggest that the intimate relationship between *Risa* and autophagy could regulate the Sirt1 nuclear plasma shuttle through complex mechanisms and then affect podocyte autophagy. It has been consistently reported that lncRNAs are less conserved than protein-coding genes [36–38]. However, evolutionary conservation may be distinct between coding and noncoding genes, such as the conservation of the lncRNA secondary structure. Meanwhile, lncRNAs from different species with conserved genomic locations may have conserved functions despite their less conserved sequences. Thus, *Risa* with partially conserved sequences in the indicated species may also have conserved biological functions, such as regulating insulin sensitivity and glucose homeostasis. It has been reported that SNP r3740051 located in human Sirt1 promoter region or predicted human *Risa* is associated with energy expenditure when food is withheld, supporting the potential effect of *Risa* on insulin sensitivity and glucose homeostasis [25]. In vivo and in vitro studies have confirmed the pathogenicity of *Risa* in DN mice, and *Risa* inhibition has a protective role in podocytes. Therefore, we tried to study the expression of *Risa* in the human body, and examined blood and urine samples of DN patients by qRT-PCR, but the results were not satisfactory. This suggested that we need to further identify and verify the relevant *Risa* SNP locus in human.

Diabetes and secondary pathologies of kidney disease are a series of pathological processes caused by changes in the body's internal environment. LncRNAs play an important role in regulating glucose homeostasis and diabetes [39]. In the present study, we discovered that *Risa* inhibition alleviated kidney damage. We speculated that this might be due to the therapeutic effect of *Risa* inhibition on kidney tissues, which improved the body's internal environment, which in turn caused alterations in uACR, Scr, and body weight. Although there was no difference in blood glucose, *Risa* inhibition treatment significantly improved all aforementioned histological and functional parameters of the kidney in the DN mice model, accompanied by restored autophagy, proving that the protective effect of *Risa* inhibition against DN might be achieved by normalizing the autophagy level in podocytes without affecting hyperglycemia. However, the limitation of this study was that it did not reveal the specific target of *Risa* in regulating Sirt1/GSK3β signaling. Further research should be performed to explore the direct downstream targets of *Risa* in podocytes, such as microRNAs, which could be translated into cellular functional effects.

## Conclusions

In conclusion, our study demonstrated that *Risa* inhibition improved the autophagy level of renal podocytes through Sirt1/GSK3β pathway, thereby ameliorating DN. These findings revealed that *Risa* was involved in a new pathway to regulate DN progression and *Risa* inhibition might be a potential strategy to improve autophagy-mediated podocyte injury and treat DN.

## Supplementary Information

**Additional file 1**.  Mouse experimental methods.

**Additional file2**: **Table S1**. Primer sequences for PCR analysis. **Table S2**. Primary antibodies for Western blotting assays. **Table S3**. Primary antibodies for immunofluorescence staining. **Fig. S1**. Dual luciferase reporter assay were applied to detect the binding site and interaction between Risa or GSK3β and predicted miRNAs.

## Data Availability

The datasets used and/or analyzed during the current study are available from the corresponding author on reasonable request.

## Abbreviations

AAV: Adeno-associated virus
Akt: Protein kinase B
AMPK: Adenosine 5′-monophosphate-activated protein kinase
ANOVA: One-way analysis of variance
*ATG*: Autophagy-related gene
ceRNAs: Competing endogenous RNAs
DAPI: 4′,6-Diamidino-2-phenylindole
DKD: Diabetic kidney disease
DN: Diabetic nephropathy
ESRD: End-stage renal disease
EX-527: Sirt1 inhibitor
FBS: Fetal bovine serum
GAPDH: Glyceraldehyde-3-phosphate dehydrogenase
GBM: Glomerular basement membrane
GSK3β: Glycogen synthase kinase 3β
HG: High glucose
HM: High mannitol
LC3B: Light chain 3 beta
LiCl: Lithium chloride
LncRNAs: Long non-coding RNAs
LV: Lentivirus
miRNA: MicroRNA
MOI: Multiplicity of infection
MPCs: Mouse podocyte cells
mTOR: Mammalian target of rapamycin
NG: Normal glucose
NIH: National institutes of health
NPHS2: Podocin
PAS: Periodic acid-schiff stain
PI: Propidium iodide
qRT-PCR: Quantitative real-time polymerase chain reaction
RIPA: Radio immunoprecipitation assay
*Risa*: LncRNA *AK044604*
Scr: Serum creatinine
SD: Standard deviation
SDS-PAGE: Sodium dodecyl sulphate–polyacrylamide gel electrophoresis
Sir2: Silent information regulator 2
Sirt1: Sirtuin 1
SNP: Single nucleotide polymorphism
SYBR: Synergy brands
SYNPO: Synaptopodin
TEM: Transmission electron microscopy
uACR: Urinary albumin-creatinine ratio
