# Supplementary material for: Down-regulation of Risa improves podocyte injury by enhancing autophagy in diabetic nephropathy
Source: Mil Med Res. 2022 May 26;9:23. doi: 10.1186/s40779-022-00385-0 (PMC9134699; doi:10.1186/s40779-022-00385-0)
Supplement: Supplementary file 1 — Additional file 1. Mouse experimental methods. [file 40779_2022_385_MOESM1_ESM.pdf]

Visual tail vein injection

These db/db mice are black, and with this breed of mice, it can be difficult to distinguish the real caudal vein by naked eye observation. The success rate of drug vein injection is less than 100% and requires expanding the caudal vein with warm water or alcohol. This can result in drug loss, insufficient drug concentration in the target organs and failure of any follow-up experiments. Therefore, achieving accurate caudal vein injection is very important for any experiment. Before the formal experiment, the author learned intravenous injection methods and skills from skilled nurses, practised on more than 10 mice every day (over more than 3 months), and improved the mouse caudal vein injection instrument by consulting the literature and patents, which led the success rate of tail vein injection in db/db mice to reach 100%. This method has repeatability, high efficiency and popularization. The improved method is an original work; thus, please provide opinions if there are deficiencies in its explanation.

Preliminary preparations

The mouse was fixed on a fixator, the tail was straightened on a venous imaging instrument, and the tail was wiped with alcohol and cotton to dilate the blood vessels. After identifying the black line on the tail, the line was gently pressed with syringe (0.5 ml) needle; if the black line disappeared directly below the needle and reappeared when the needle was withdrawn, this is the real caudal vein. We chose only straight and thick veins for injection. We fixed the tail with the left hand, held the syringe with the right hand, and placed the right hand on the left hand to avoid any hand tremor. With the bevel side of the needle tip pointed upwards, the entry needlepoint was located 1/3 or 1/4 posterior to the tail tip; as the skin in this location is thinner, the blood vessel is clear, and the needle is easier to introduce. After the injection, bleeding was stopped with medical alcohol cotton for approximately 1 min.

Key step 1: Use a vein imaging instrument with a heating function to visualize the caudal vein injection process.

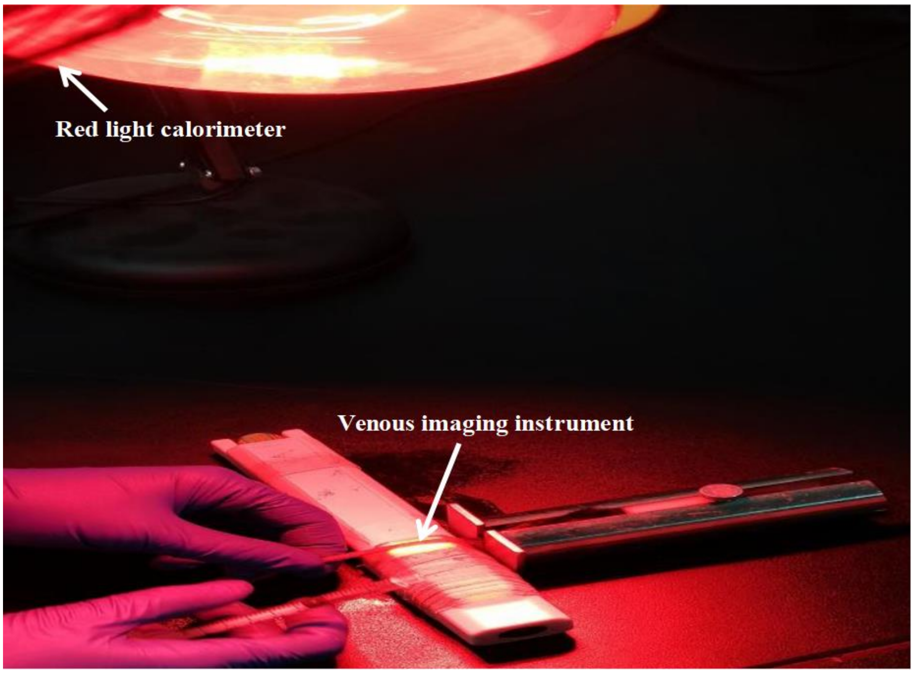

Key step 2: The mouse was placed into the fixator, and the mouse tail was straightened on the light source of the vein imaging instrument.

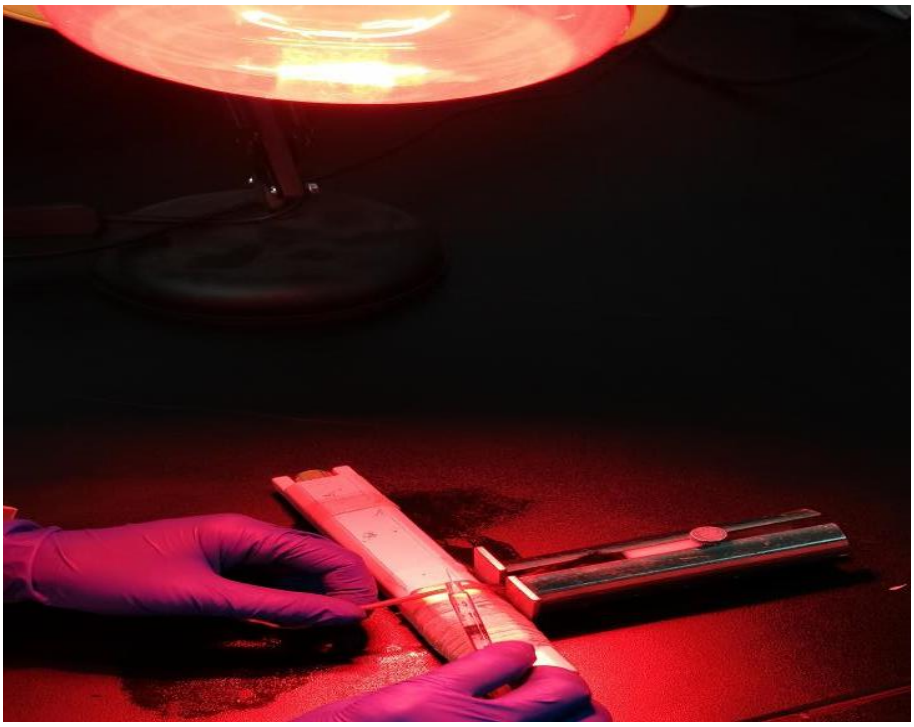

Key step 3: The drug was drawn with a 0.5 ml syringe, the mouse tail was fixed with the left hand, and the black line (the real vein) was observed with a vein imager (shown

by the yellow arrow). The black line was gently pressed with a needle, the line disappeared directly below the needle (white arrow) and then reappeared when the needle was withdrawn.

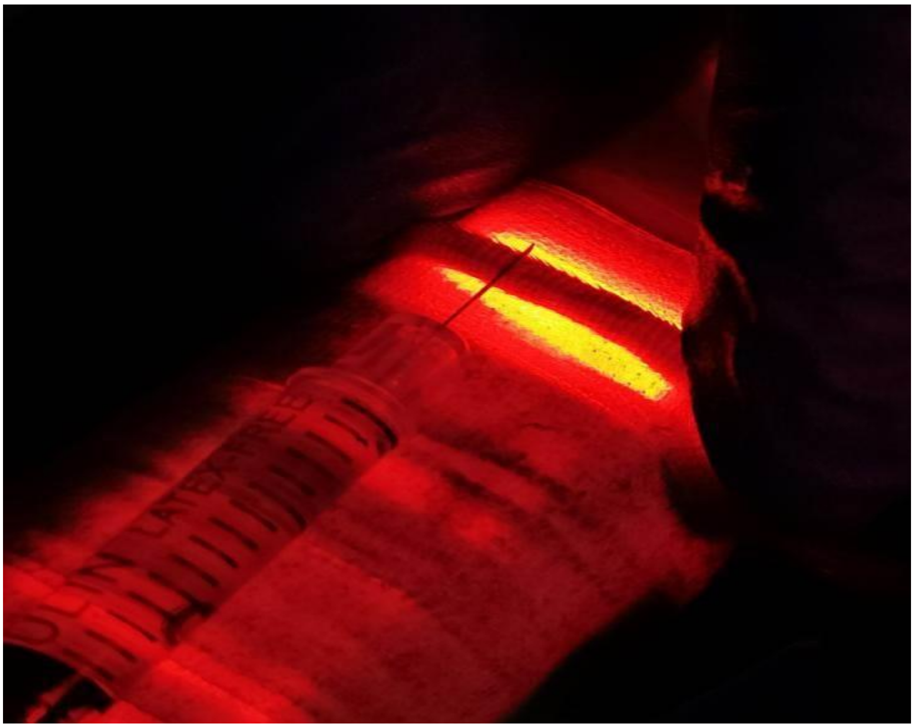

Key step 4: A straight and thick vein was selected for injection. The tail was fixed with the left hand, the syringe was held with the right hand, and the right hand was placed on the left hand to avoid any hand tremors. The inclined plane of the needle points upward and is kept 15 degrees from the horizontal plane. The entry needle point is located 1/3 or 1/4 posterior to the tail tip. After approximately 3-5 mm is inserted into the black line (caudal vein), drug injection is unobstructed without resistance and the black line suddenly disappears, indicating that the caudal vein injection is successful. After the injection, the bleeding was stopped with medical alcohol cotton for approximately 1 min.

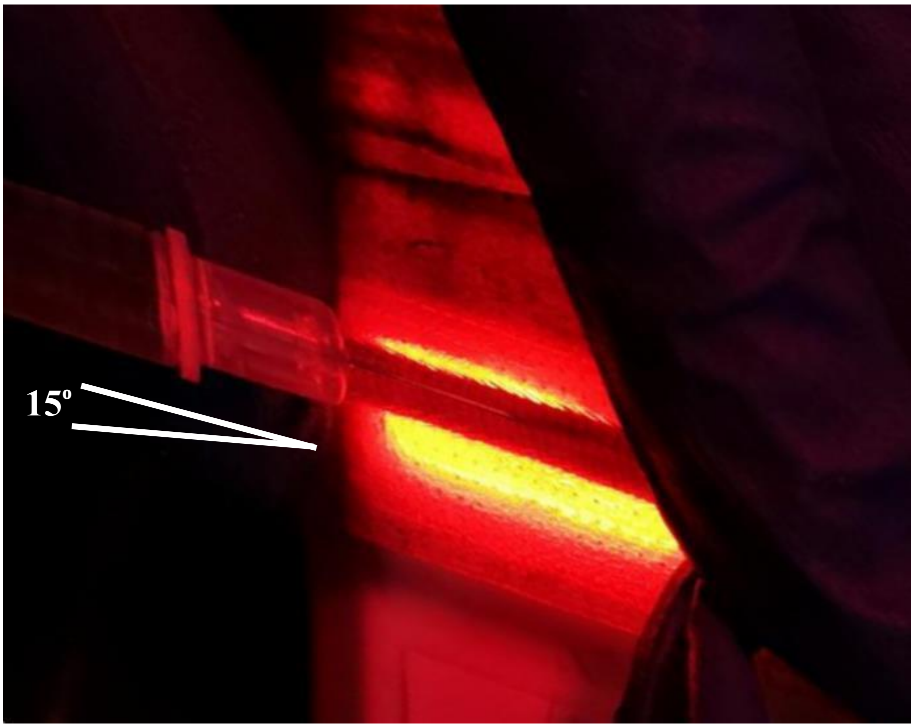

Treatment of renal tissue specimens

First, the renal tissue was perfused and separated. Then, the tissues were cut into parts for different uses. The uppermost renal tissues were cut into 1 × 1 × 1 mm^3 pieces, stored in electron microscope solution (2.5% glutaraldehyde fixed solution), and sent to the electron microscope room to process the specimens, where the ultrastructure and autophagosomes were observed. The upper renal tissues were stored in light microscope solution (10% neutral formalin) and sent to the light microscope room for PAS staining, and the morphological changes were observed. The intermediate kidney tissues were placed in embedding agent, subcooled in liquid nitrogen, and then transferred to -80°C for preservation. The lower kidney tissues were placed in sterile enzyme-free EP tubes and ordinary EP tubes, subcooled in liquid nitrogen, and then transferred to -80°C for preservation.

Blood glucose measurement

The tail of the mice was washed with warm water or alcohol, tail vein blood was collected under a vein imager, blood glucose was measured three times with a blood glucose metre, and the average value was calculated.

Cardiac blood collection method

The mice were fixed in place after anaesthesia. After fully exposing the heart by dissection, we punctured the right ventricle with a 1 ml syringe, extracted the blood slowly, placed the blood into a 1.5 ml EP tube, centrifuged the EP tube at 4°C and 3000 rpm for 10 min, extracted the supernatant, and sent blood samples to the biochemical laboratory of the First Affiliated Hospital of Zhengzhou University. Then, the blood creatinine levels were measured by an automatic biochemical detector.

The total blood volume of mice is only approximately 1 - 3 ml; thus, each step of the operation is crucial to the success of the heart blood collection and follow-up experiments (such as serum creatinine and qRT-PCR). Before the formal experiment, the author mastered the cardiac anatomy of the mice. The volume of cardiac blood collected was approximately 1 ml/mouse using the described method. The method is reproducible. This method is an original work, and please provide guidance if there are any deficiencies in its explanation.

Step 1: After dissecting the mouse, the thorax was fully exposed and the heart position was adjusted to the median line of the mouse (red arrow) with tweezers.

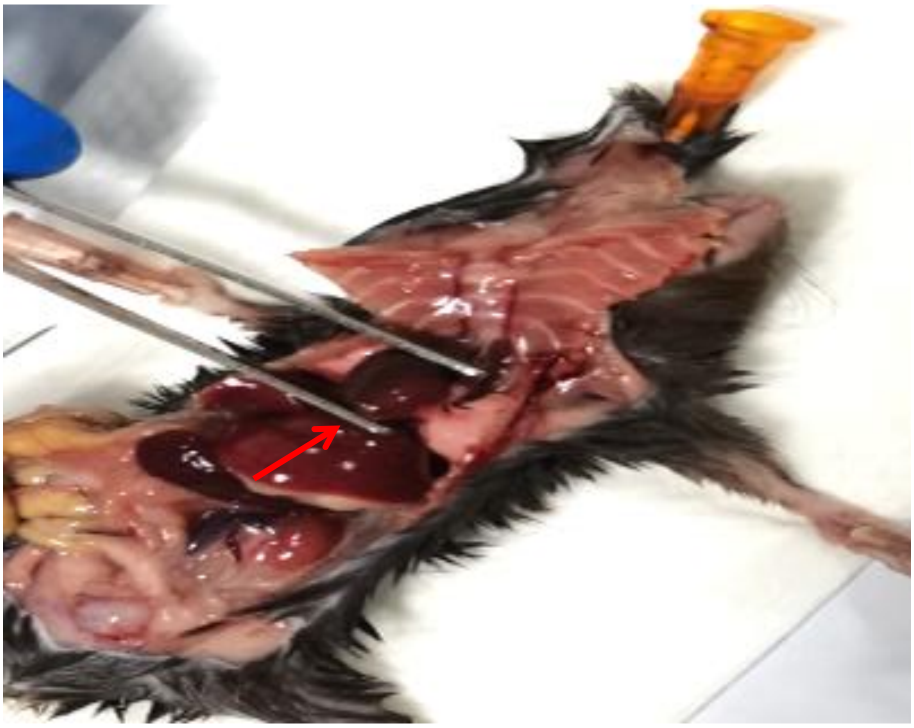

Step 2: The heart was carefully observed. The darker side is venous blood and the lower part corresponds to the right ventricle (red arrow), while the lighter side is arterial blood and the lower part corresponds to the left ventricle (yellow arrow).

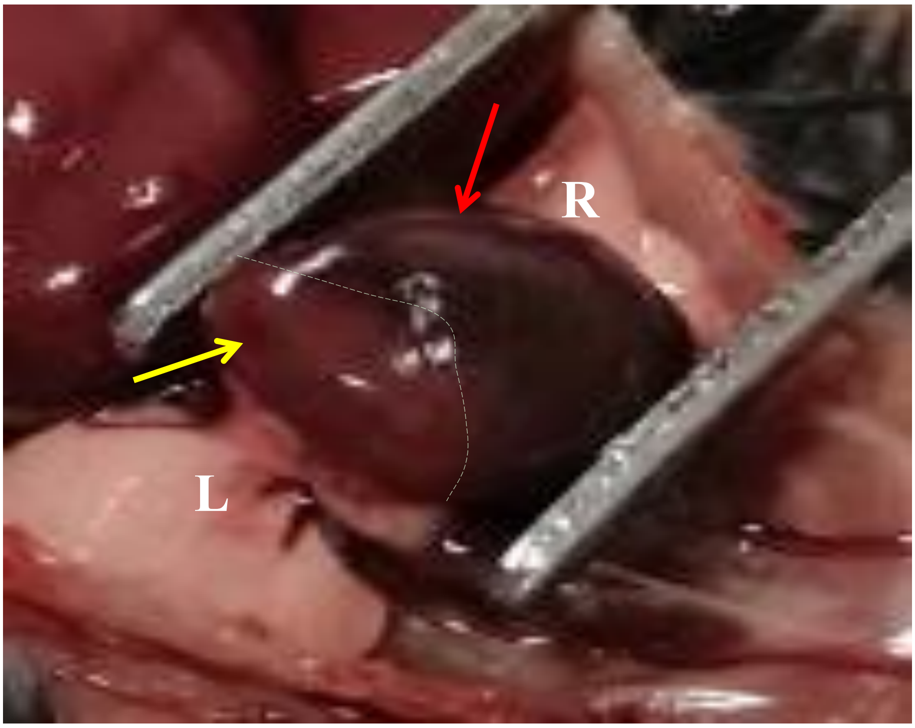

Step 3: After fixing both hands on the table (green arrow) to prevent any hand tremor from affecting the blood collection, the inclined plane of a 1 ml syringe needle was pointed upwards and kept 15 degrees from the horizontal plane (red arrow). The needle was inserted into the right ventricle at a depth of approximately 4 mm without resistance, and the needle tip was slightly biased towards the distal end of the right ventricle to prevent perforation of the ventricular septum, which causes blood collection failure.

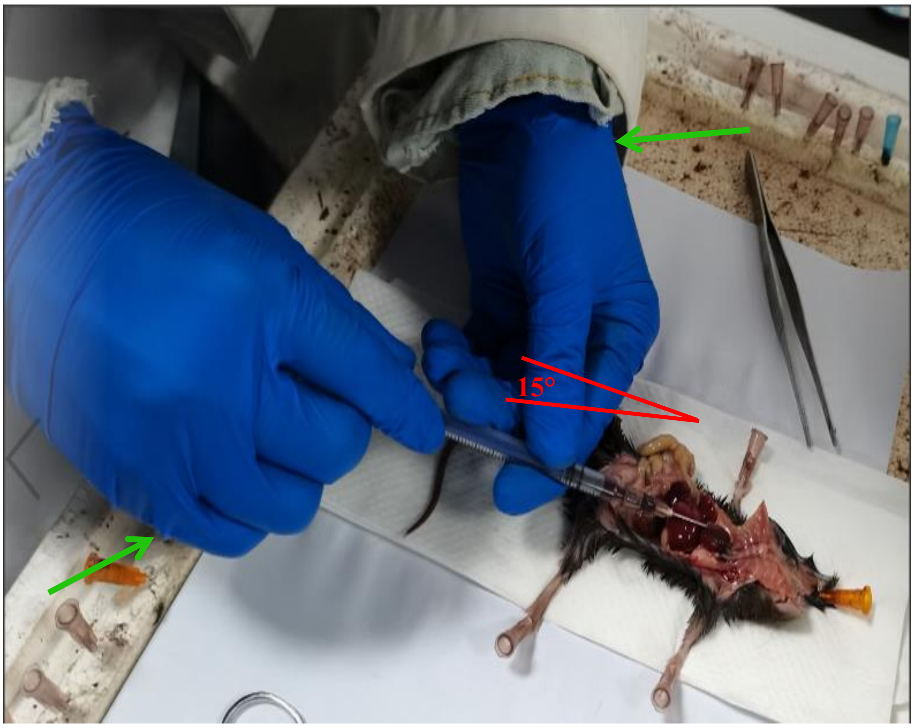

Step 4: The blood was drawn with gentle force, and collecting more than 0.5 ml of blood was considered successful. The needle tip can continue to enter the right atrium to collect additional blood.

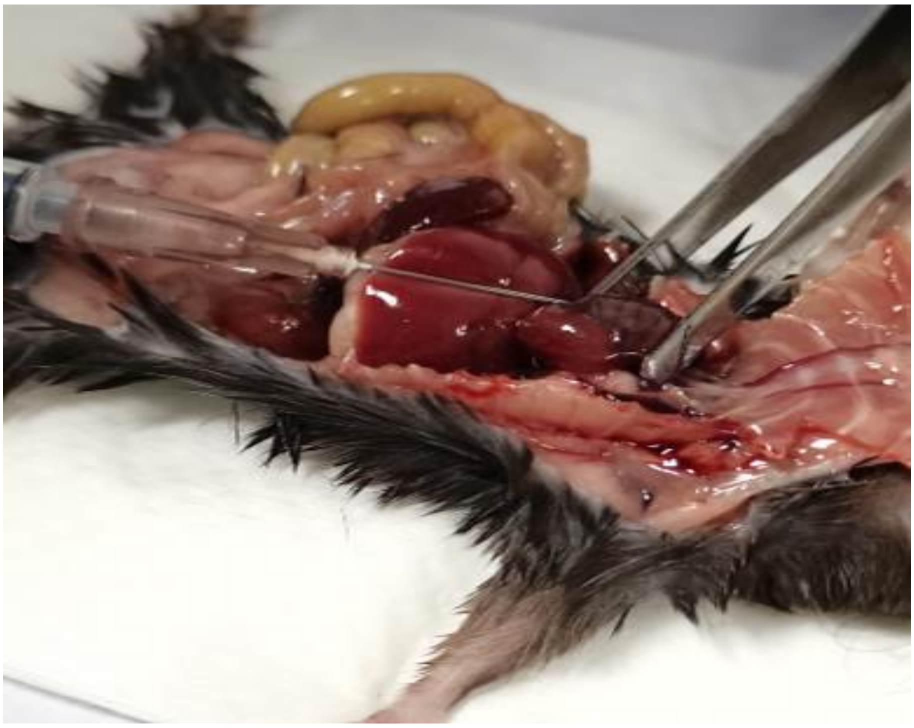

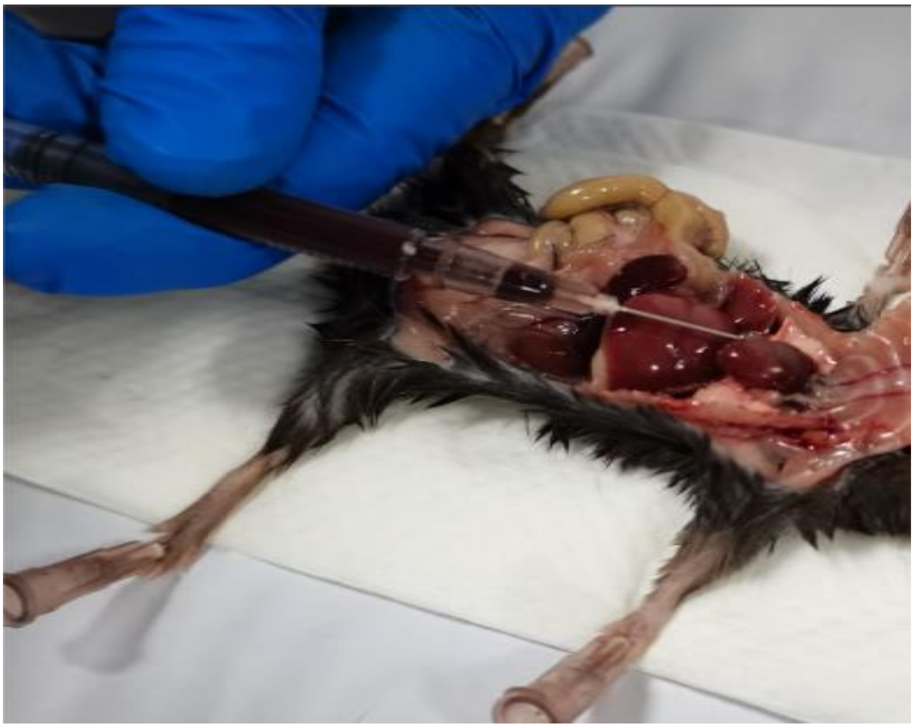

After collecting blood from the heart, the blood sample was placed in the EP tube and centrifuged at 3000 rpm for 10 min at 4°C. The supernatant was extracted into a new EP tube, and the supernatant and blood cells were stored at -80°C.

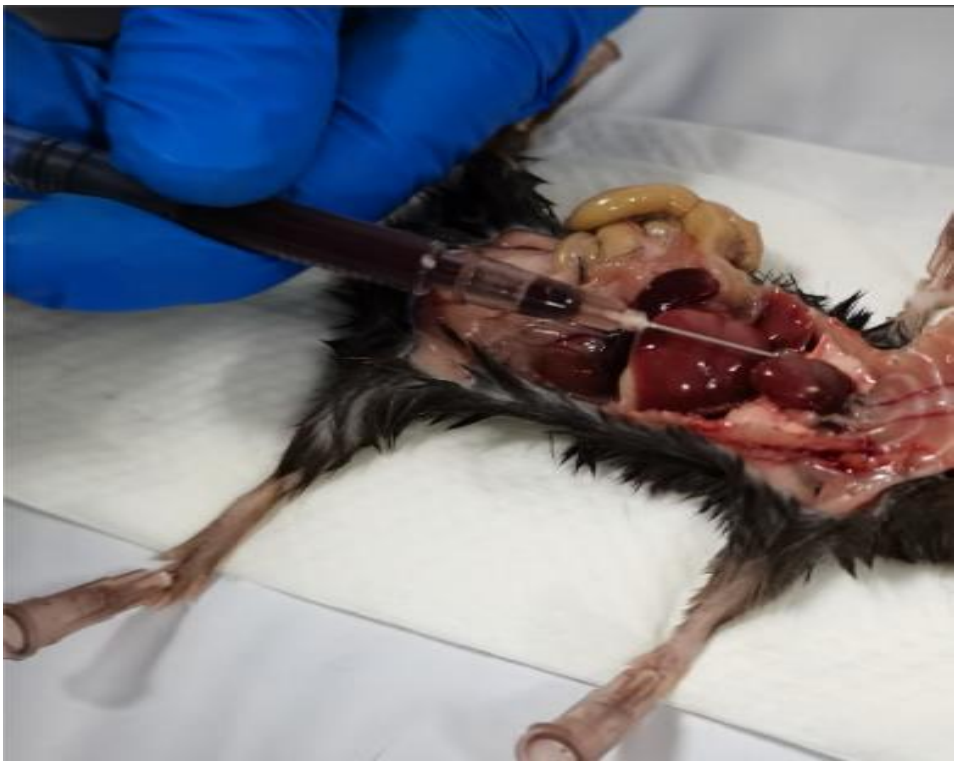

Urine collection and determination

Water was withdrawn from the mice during the collection stage. The mice were placed in clean PCR boxes and their urine was placed in EP tubes. The tubes were centrifuged at 12,000 rpm for 10 min at 4°C, and the supernatant was collected and sent to the Central Laboratory of the First Affiliated Hospital of Zhengzhou University. Then, the urine ACR was measured by an automatic analyzer.
