# Supplementary material for: Down-regulation of Risa improves podocyte injury by enhancing autophagy in diabetic nephropathy
Source: Mil Med Res. 2022 May 26;9:23. doi: 10.1186/s40779-022-00385-0 (PMC9134699; doi:10.1186/s40779-022-00385-0)
Supplement: Supplementary file 2 — Additional file2: Table S1. Primer sequences for PCR analysis. Table S2. Primary antibodies for Western blotting assays. Table S3. Primary antibodies for immunofluorescence staining. Fig. S1. Dual luciferase reporter assay were applied to detect the binding site and interaction between Risa or GSK3β and predicted miRNAs. [file 40779_2022_385_MOESM2_ESM.pdf]

Table S1 Primer sequences for PCR analysis

**Gene (mouse)**	**Primer sequence (5′–3′)**	
Risa	Forward	TCTGGAGAGCCCAACCT
	Reverse	TCCTTCAAACGCGAGAGAG
Beclin-1	Forward	GAGTGGAATGAAATCAATGCTGC
	Reverse	TTTCCACCTCTTCTTTGAACTGC
LC3B	Forward	CCGTCCGAGAAGACCTTCAA
	Reverse	TCTTGCGGCAGGAGAACCTA
GAPDH	Forward	CCTCGTCCCGTAGACAAAATG
	Reverse	TGAGGTCAATGAAGGGGTCGT

Risa lncRNA *AK044604*, *LC3B* light chain 3 beta, *GAPDH* glyceraldehyde-3-phosphate dehydrogenase

Table S2 Primary antibodies for Western blotting assays

**Name**	**Corporation**
Anti-Beclin 1 antibody	Abcam, Servicebio
Anti-Desmin antibody	Abcam, Servicebio
Anti-GAPDH antibody	Good here
Anti-GSK3β (phospho Ser9) antibody	CST
Anti-GSK3β antibody	CST, Servicebio
Anti-LC3B antibody	Sigma
Anti-Nephrin antibody	Abcam
Anti-NPHS2 antibody	Abcam
Anti-Sirt1 antibody	Bioss
Anti-Sirt1 (phospho Ser27) antibody	Bioss
Anti-SQSTM1/p62 antibody	Servicebio
Anti-WT1 antibody	CST, Servicebio

GAPDH glyceraldehyde-3-phosphate dehydrogenase, *GSK3β* glycogen synthase kinase 3β, *LC3B* light chain 3 beta, *NPHS2* podocin, *Sirt1* sirtuin 1

Table S3 Primary antibodies for immunofluorescence staining

**Name**	**Corporation**
Anti-GSK3β (phospho Ser9) antibody	CST
Anti-LC3B antibody	Sigma, CST
Anti-Nephrin antibody	Servicebio, Abcam
Anti-Sirt1 (phospho Ser27) antibody	Bioss
Anti-SQSTM1/p62 antibody	Servicebio
Anti-Synaptopodin antibody	Santa
Anti-WT1 antibody	CST

GSK3β glycogen synthase kinase 3β, *LC3B* light chain 3 beta

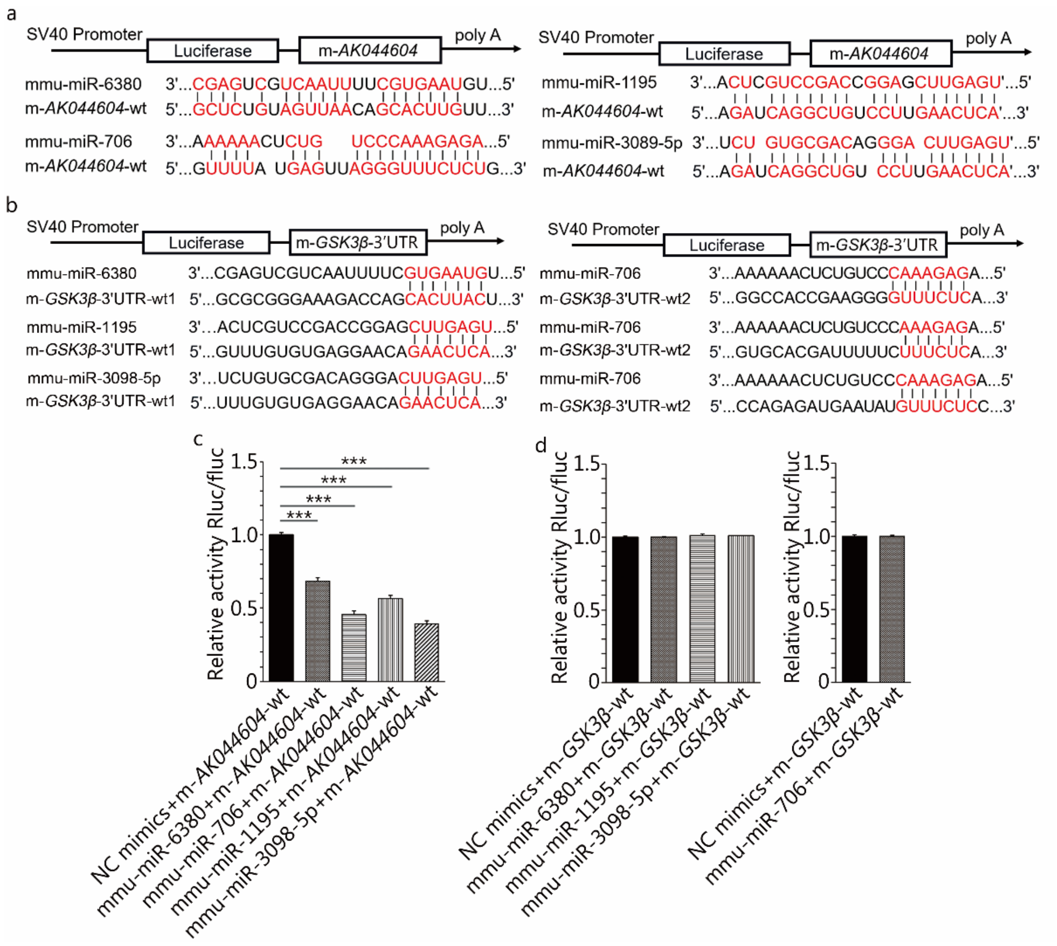

Fig. S1 Dual luciferase reporter assay were applied to detect the binding site and interaction between *Risa* or *GSK3β* and predicted miRNAs. **a** Schematic of mmu-miR-6380, mmu-miR-706, mmu-miR-1195 and mmu-miR-3098-5p binding to m-*AK044604* targets. **b** Schematic of mmu-miR-6380, mmu-miR-1195, mmu-miR-3098-5p and mmu-miR-706 binding to m-*GSK3β*-3'UTR targets. **c** The interaction between mmu-miR-6380, mmu-miR-706, mmu-miR-1195, mmu-miR-3098-5p and m-*AK044604*. **d** The interaction between mmu-miR-6380, mmu-miR-1195, mmu-miR-3098-5p, mmu-miR-706 and m-*GSK3β*-3'UTR. Values are expressed as mean ± SD of three independent experiments. $^{***}P < 0.001$. *Risa* lncRNA *AK044604*, *GSK3β* glycogen synthase kinase 3β, *NC* normal control
